# Supplementary material for: Maternal Diet during Pregnancy Alters the Metabolites in Relation to Metabolic and Neurodegenerative Diseases in Young Adult Offspring
Source: Int J Mol Sci. 2024 Oct 14;25(20):11046. doi: 10.3390/ijms252011046 (PMC11508017; doi:10.3390/ijms252011046)
Supplement: Supplementary file 1 [file ijms-25-11046-s001.zip › ijms-3224788-supplementary.pdf]

Maternal Diet during Pregnancy Alters the Metabolites in Relation to Metabolic and Neurodegenerative Diseases in Young Adult Offspring

Soo-Min Kim, Songjin Oh, Sang Suk Lee, Sunwha Park, Young-Min Hur, AbuZar Ansari, Gain Lee, Man-Jeong Paik, Young-Ah You and Young Ju Kim

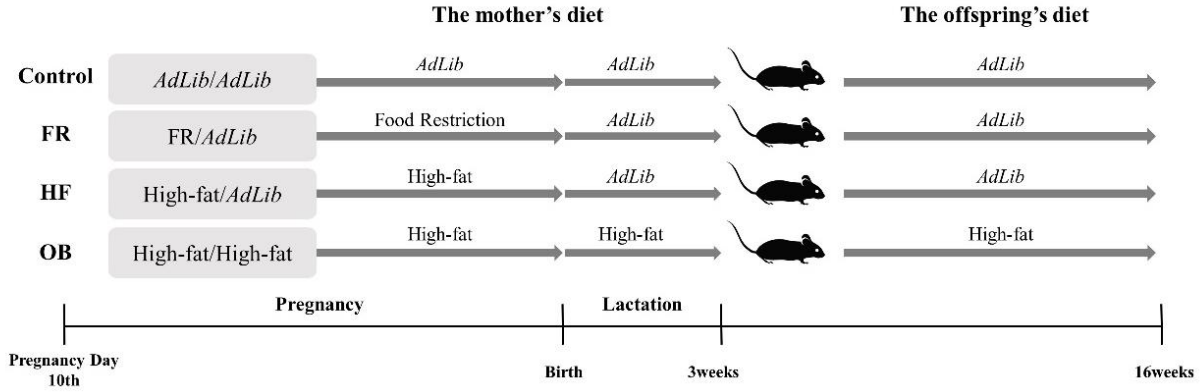

Figure S1. Study design

Sprague-Dawley (SD) rats were divided into four groups by maternal diet during pregnancy and lactation: (I) Control group (control, *Ad-Libitum*/*Ad-Libitum*), (II) Food-restriction group (FR, 50% Food-restriction/*Ad-Libitum*), (III) High-fat group (HF, 45% high-fat/*Ad -Libitum*), and (IV) Obese group (OB, 45% high-fat/45% high-fat). After lactation, the offspring in Control, FR and HF groups were fed chow diet until 16-week-old, and the offspring in OB group was given a 45% high-fat diet. FR, Food-restriction; HF, High-fat diet; OB, Obese group.

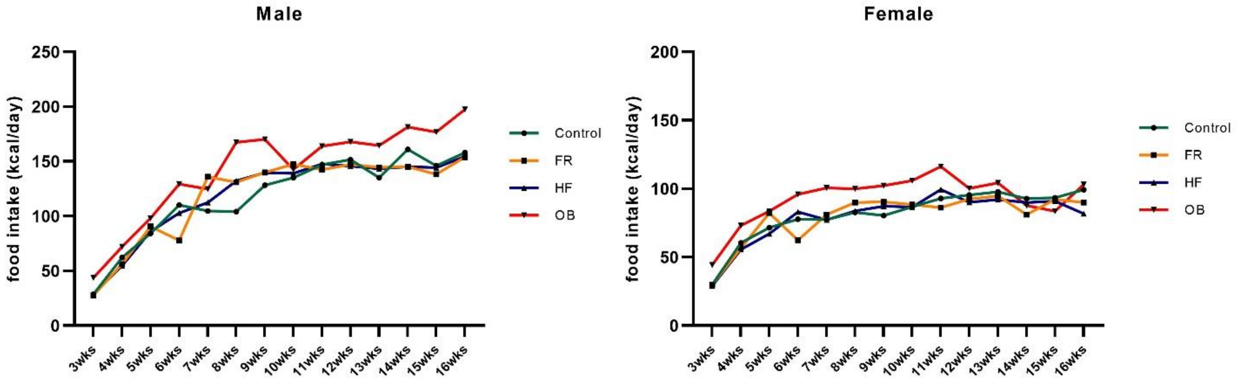

Figure S2. Comparison of the food intake among the four groups in the offspring after the lactation period.

The amount of food consumed per day from 3 weeks of age to 16 weeks of age in the control group (green line), FR group (yellow line), HF group (blue line) and OB group (red line). FR, Food-restriction; HF, High-fat diet; OB, Obese group.

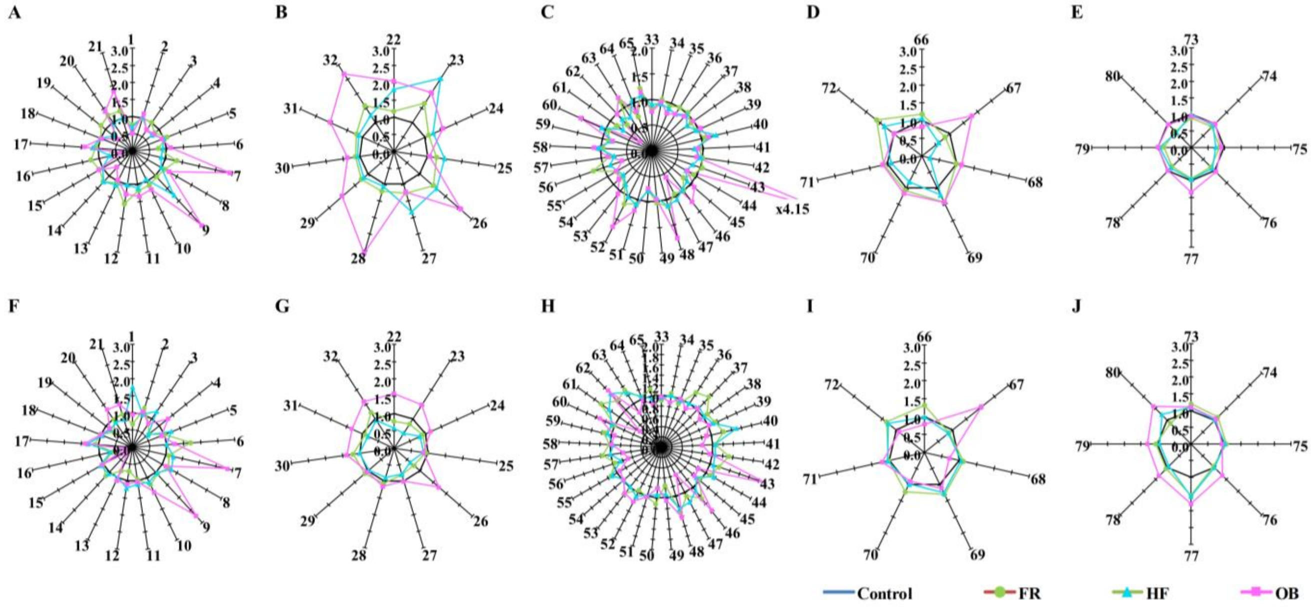

Figure S3. Star symbol patterns in male and female rat offspring of FR, HF, and OB groups.

A total of 80 metabolites, including 21 organic acids, 11 fatty acids, 33 amino acids, 7 kynurenic acids, and 8 nucleosides, were identified in the plasma of FR, HF, and OB males and females using GC-MS/MS and LC-MS/MS. The concentrations of the 80 metabolites in male and female offspring of the FR, HF, and OB groups are presented in Supplementary Tables 2 and 3, respectively. Organic acids (A: male; F: female), fatty acids (B: male; G: female), amino acids (C: male; H: female), kynurenic acids (D: male; I: female), and nucleosides (E: male; J: female).

A

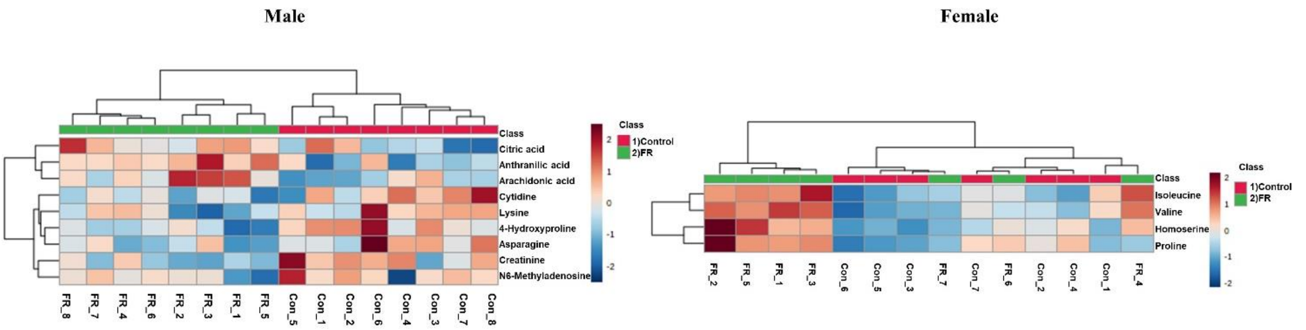

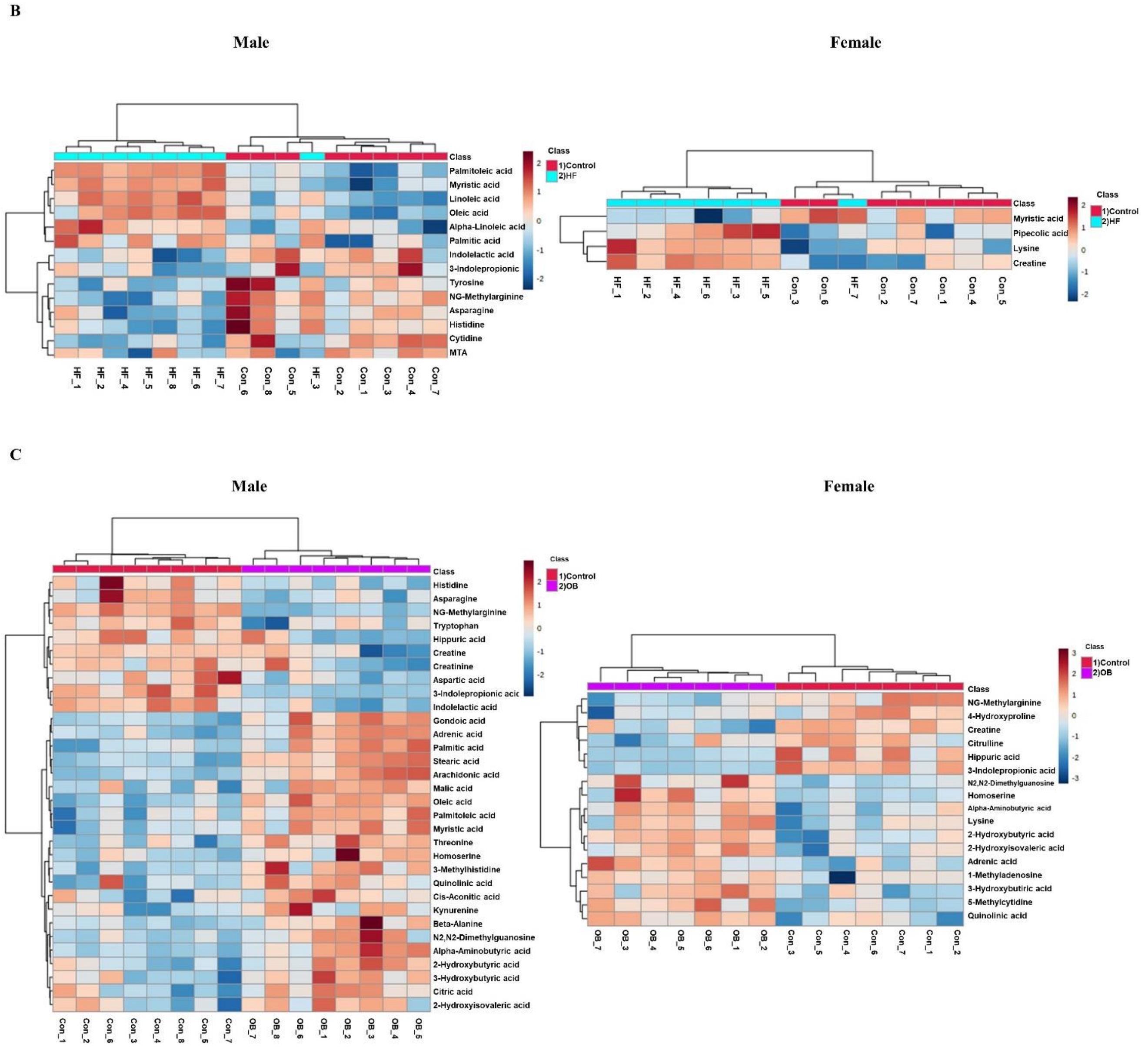

Figure S4. Hierarchical clustering heatmap analysis of significant different metabolites in the FR, HF and OB groups

(A) The control and FR male groups clustered in heatmap analysis with 9 metabolites ($p < 0.05$), but FR female groups did not cluster in heatmap analysis with 4 metabolites ($p < 0.05$). (B) With the exception of one case, the control and HF male groups clustered in the heat map analysis with the 14 metabolites ($p < 0.05$) and the control and HF female groups clustered in the heat map analysis with the 4 metabolites ($p < 0.05$). (C) The control and OB male groups clustered in heat map analysis with the 32 metabolites ($p < 0.05$) and the control and OB female groups clustered in heat map analysis with the 17 metabolites ($p < 0.05$) except for one case.

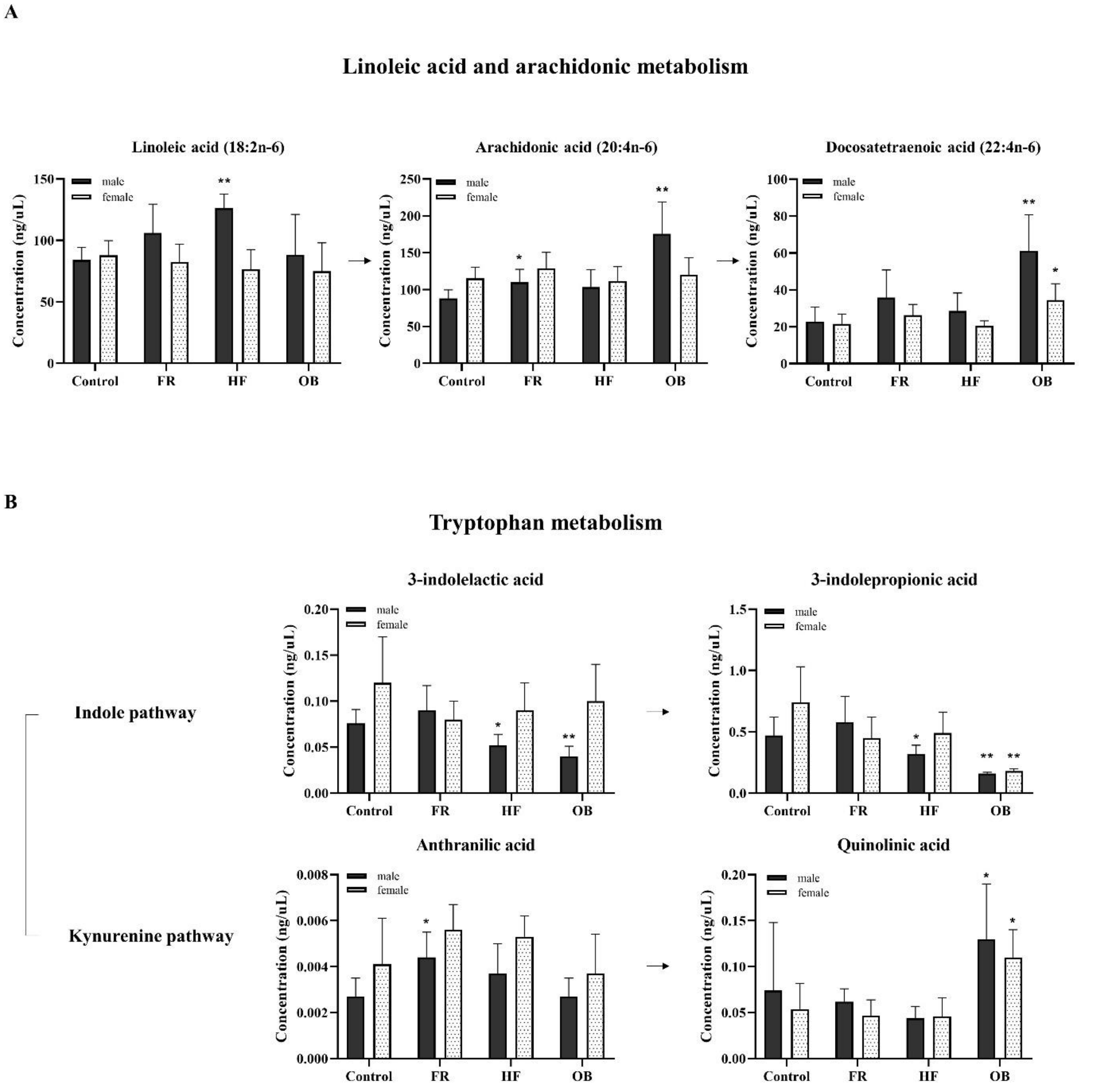

Figure S5. Comparison of metabolites associated with metabolic pathway in offspring groups.

We compared the metabolites involved in the metabolism of tryptophan, arachidonic acid, and linoleic acid, which were prominent in the pathway analysis of the male groups compared to that in the controls. (A) Linoleic acid (18:2n-6), arachidonic acid (20:4n-6), and docosatetraenoic acid (22:4n-6), which are omega-6 fatty acids, were measured using gas chromatography tandem mass spectrometry (GC-MS/MS). (B) Metabolites involved in tryptophan metabolism; indole pathway (3-indolelactic acid and 3-indolepropionic acid (IPA)), and kynurenine pathway (anthranilic acid and quinolinic acid) were measured using gas chromatography tandem mass spectrometry (GC-MS/MS). * $p < 0.05$ versus Control, ** $p < 0.001$ versus Control.

Table S1. Comparison of maternal plasma carbohydrate and lipid profiles

Variables	Control<br>(Mean ± SD)	FR<br>(Mean ± SD)	HF<br>(Mean ± SD)	OB<br>(Mean ± SD)
Glucose (mg/dL)	144.25 ± 39.96	166.25 ± 29.41	146.75 ± 48.40	144.75 ± 39.07
Total cholesterol (mg/dL)	56.25 ± 14.22	84.50 ± 14.06 *	80.50 ± 18.63	51.00 ± 15.03
Triglyceride (mg/dL)	41.75 ± 22.60	66.50 ± 29.89	83.25 ± 39.78	54.75 ± 14.31
Insulin (mg/mL)	0.19 ± 0.18	0.60 ± 0.25 *	0.19 ± 0.26	0.15 ± 0.16
HOMA-IR	1.87 ± 2.01	5.95 ± 2.02 *	2.24 ± 3.64	1.61 ±2.08

Data are expressed as mean ± SD. * $p < 0.05$ versus Control, ** $p < 0.001$ versus Control. HOMA-IR, homeostatic model assessment for insulin resistance; FR, food restriction; HF, high-fat diet; OB, obese group.

Table S2. Levels and P-values of metabolites in plasma of male rat models

No.	Metabolite	Concentration (ng/μL, Mean ± SD)				Normalized value[a]			P-value[b]		
		Control	FR	HF	OB	Control vs FR	Control vs HF	Control vs OB	Control vs FR	Control vs HF	Control vs OB
**Organic acid**											
1	Pyruvic acid	23.2 ± 16.8	17.4 ± 8.5	15.6 ± 11.1	11.5 ± 8.4	0.75	0.67	0.50	0.645	0.195	0.161
2	Acetoacetic acid	31.4 ± 11.2	31.1 ± 8.5	32.3 ± 5.7	35.4 ± 4.2	0.99	1.03	1.13	0.798	0.798	0.878
3	Lactic acid	200.5 ± 90.2	200.5 ± 77.1	149.4 ± 57.2	147.1 ± 64.7	1.00	0.75	0.73	0.959	0.382	0.195
4	Glycolic acid	2.2 ± 1.1	1.8 ± 1.7	1.6 ± 1.0	1.7 ± 0.7	0.83	0.74	0.79	0.878	0.798	0.721
5	Phenylacetic acid	0.18 ± 0.03	0.19 ± 0.05	0.18 ± 0.02	0.16 ± 0.02	1.09	0.99	0.93	0.798	0.798	0.382
6	Oxalic acid	3.3 ± 1.2	2.5 ± 1.2	2.9 ± 1.2	3.7 ± 1.2	0.76	0.89	1.12	0.195	0.574	0.574
7	2-Hydroxybutyric acid	1.3 ± 0.7	1.7 ± 1.1	1.1 ± 0.3	3.8 ± 2.2	1.33	0.87	2.93	0.505	0.959	0.0070
8	3-Hydroxypropionic acid	0.51 ± 0.17	0.56 ± 0.21	0.50 ± 0.13	0.63 ± 0.10	1.10	0.98	1.24	0.721	0.645	0.130
9	3-Hydroxybutyric acid	99.5 ± 76.5	104.6 ± 51.6	178.1 ± 95.9	296.8 ± 220.7	1.05	1.79	2.98	0.645	0.235	0.0070
10	2-Hydroxyisovaleric acid	0.27 ± 0.06	0.31 ± 0.09	0.25 ± 0.03	0.34 ± 0.06	1.14	0.94	1.26	0.645	0.574	0.050
11	Succinic acid	2.6 ± 0.7	3.0 ± 0.9	2.9 ± 1.2	3.5 ± 1.4	1.18	1.12	1.37	0.279	0.721	0.161
12	Fumaric acid	0.38 ± 0.12	0.60 ± 0.30	0.38 ± 0.22	0.49 ± 0.15	1.58	1.01	1.29	0.161	0.878	0.279
13	α-Ketoglutaric acid	8.5 ± 1.7	9.1 ± 1.7	9.6 ± 2.7	8.2 ± 1.8	1.06	1.13	0.96	0.442	0.328	0.798
14	Hippuric acid	0.41 ± 0.10	0.52 ± 0.14	0.51 ± 0.25	0.28 ± 0.12	1.25	1.23	0.66	0.161	0.798	0.021
15	Malic acid	6.4 ± 0.5	7.1 ± 1.0	6.4 ± 0.9	7.2 ± 0.5	1.11	1.01	1.14	0.235	1.000	0.015
16	3-Indolepropionic acid	0.47 ± 0.15	0.58 ± 0.21	0.32 ± 0.07	0.16 ± 0.01	1.24	0.67	0.35	0.161	0.028	0.00016
17	*cis*-Aconitic acid	0.057 ± 0.017	0.061 ± 0.021	0.066 ± 0.010	0.080 ± 0.022	1.07	1.16	1.40	0.721	0.279	0.050
18	3-Indolecarboxylic acid	0.088 ± 0.007	0.087 ± 0.008	0.087 ± 0.006	0.084 ± 0.005	0.99	0.99	0.95	0.798	0.878	0.328
19	3-Indolelactic acid	0.076 ± 0.015	0.090 ± 0.027	0.052 ± 0.012	0.040 ± 0.011	1.17	0.69	0.53	0.328	0.0047	0.00062
20	Citric acid	6.0 ± 1.5	7.6 ± 1.2	5.8 ± 0.8	8.3 ± 1.5	1.27	0.97	1.39	0.028	0.878	0.010
21	Isocitric acid	1.1 ± 0.2	1.3 ± 0.3	1.0 ± 0.2	1.9 ± 1.4	1.21	0.99	1.81	0.161	0.798	0.065
**Fatty acid**											
22	Myristic acid	1.3 ± 0.4	1.5 ± 0.8	2.3 ± 0.5	2.7 ± 0.8	1.17	1.79	2.04	0.798	0.0030	0.0030
23	Palmitoleic acid	3.7 ± 1.3	6.1 ± 3.9	9.3 ± 2.2	7.5 ± 2.0	1.66	2.52	2.03	0.382	0.0011	0.0011
24	Palmitic acid	73.3 ± 11.1	82.2 ± 12.6	92.7 ± 9.4	116.2 ± 22.9	1.12	1.26	1.59	0.382	0.010	0.0019
25	Linoleic acid	84.2 ± 10.2	106.0 ± 23.6	126.3 ± 11.4	88.3 ± 32.9	1.26	1.50	1.05	0.105	0.00016	0.574
26	Oleic acid	25.3 ± 6.2	38.7 ± 18.6	41.9 ± 8.5	65.5 ± 15.5	1.53	1.65	2.59	0.105	0.0019	0.00016
27	α-Linolenic acid	2.9 ± 0.9	3.7 ± 1.3	5.3 ± 1.3	3.6 ± 1.5	1.28	1.84	1.24	0.328	0.0011	0.442
28	Stearic acid	22.8 ± 5.2	27.7 ± 9.6	24.5 ± 7.9	70.5 ± 19.7	1.22	1.07	3.09	0.382	0.328	0.00016
29	Arachidonic acid	88.3 ± 11.3	110.2 ± 17.4	103.4 ± 23.8	175.7 ± 43.0	1.25	1.17	1.99	0.010	0.328	0.00016
30	Eicosadienoic acid	2.3 ± 1.1	2.5 ± 1.0	2.4 ± 1.0	3.1 ± 0.8	1.12	1.07	1.36	0.442	0.645	0.105
31	Gondoic acid	0.46 ± 0.07	0.55 ± 0.12	0.51 ± 0.04	0.93 ± 0.25	1.19	1.12	2.04	0.130	0.130	0.00031
32	Docosatetraenoic acid	22.8 ± 7.8	35.8 ± 15.0	28.5 ± 9.8	61.0 ± 19.7	1.57	1.25	2.67	0.105	0.279	0.00062
**Amino acid**											
33	Creatinine	2.1 ± 0.3	1.6 ± 0.2	1.9 ± 0.2	1.6 ± 0.6	0.79	0.91	0.76	0.015	0.382	0.050
34	Phenylalanine	13.9 ± 2.6	14.3 ± 1.2	12.9 ± 1.6	13.5 ± 1.6	1.03	0.93	0.97	0.645	0.645	0.959
35	Tryptophan	9.3 ± 1.0	8.7 ± 0.9	8.2 ± 1.0	7.3 ± 1.5	0.93	0.88	0.78	0.279	0.161	0.007
36	Leucine	24.8 ± 4.6	24.9 ± 3.0	21.0 ± 5.2	20.9 ± 3.4	1.00	0.85	0.84	0.798	0.105	0.279
37	Isoleucine	14.6 ± 2.7	15.0 ± 1.3	14.3 ± 2.5	13.4 ± 2.6	1.03	0.98	0.92	0.442	0.959	0.574
38	Methionine	9.9 ± 1.9	9.3 ± 0.6	8.3 ± 0.9	9.8 ± 1.4	0.94	0.84	0.99	0.721	0.161	0.878
39	Proline	18.0 ± 2.6	17.1 ± 1.1	17.1 ± 2.3	18.8 ± 2.5	0.95	0.95	1.04	0.505	0.505	0.645

40	Pipecolic acid	0.48 ± 0.08	0.51 ± 0.10	0.62 ± 0.10	0.55 ± 0.31	1.06	1.29	1.14	0.574	0.065	0.798
41	Tyrosine	19.3 ± 3.6	17.9 ± 2.3	16.0 ± 1.9	17.4 ± 2.3	0.93	0.83	0.90	0.574	0.050	0.505
42	Valine	28.5 ± 5.0	28.4 ± 1.7	27.5 ± 6.7	25.2 ± 4.8	1.00	0.96	0.88	0.574	0.505	0.382
43	α-Aminobutyric acid	0.83 ± 0.16	0.86 ± 0.17	0.70 ± 0.10	2.9 ± 1.6	1.04	0.84	3.43	0.878	0.161	0.00016
44	Alanine	66.3 ± 16.6	62.5 ± 6.9	51.3 ± 9.7	52.8 ± 9.3	0.94	0.77	0.80	0.645	0.083	0.161
45	4-Hydroxyproline	3.0 ± 0.3	2.4 ± 0.2	2.7 ± 0.5	3.3 ± 0.3	0.81	0.92	1.10	0.0030	0.279	0.105
46	Homoserine	27.5 ± 2.7	27.3 ± 2.8	26.6 ± 3.1	35.0 ± 7.0	0.99	0.96	1.27	1.000	0.721	0.0070
47	Creatine	56.4 ± 5.9	53.7 ± 6.3	60.7 ± 13.9	33.4 ± 21.5	0.95	1.08	0.59	0.279	1.000	0.021
48	Threonine	213.4 ± 62.9	243.3 ± 71.9	239.3 ± 69.2	379.3 ± 91.5	1.14	1.12	1.78	0.442	0.505	0.0019
49	α-Aminoadipic acid	0.22 ± 0.04	0.23 ± 0.04	0.20 ± 0.03	0.21 ± 0.06	1.03	0.93	0.96	0.878	0.328	0.328
50	Glycine	30.7 ± 9.2	24.0 ± 3.5	24.3 ± 4.4	22.9 ± 6.4	0.78	0.79	0.75	0.279	0.279	0.083
51	Glutamic acid	18.2 ± 4.5	19.7 ± 4.1	19.9 ± 3.5	22.3 ± 2.7	1.09	1.10	1.22	0.279	0.235	0.083
52	β-Alanine	0.26 ± 0.03	0.31 ± 0.11	0.26 ± 0.03	0.43 ± 0.19	1.18	1.02	1.67	0.328	0.959	0.0030
53	Serine	28.2 ± 5.8	24.0 ± 2.1	23.5 ± 4.0	30.3 ± 2.8	0.85	0.83	1.07	0.130	0.083	0.195
54	Glutamine	90.8 ± 40.3	71.2 ± 10.5	66.4 ± 10.8	77.6 ± 8.9	0.78	0.73	0.85	0.161	0.105	0.645
55	Asparagine	11.1 ± 2.0	9.0 ± 1.1	8.8 ± 1.8	9.0 ± 1.0	0.81	0.79	0.81	0.021	0.028	0.021
56	Aspartic acid	0.9 ± 0.3	1.1 ± 0.4	0.79 ± 0.26	0.56 ± 0.12	1.21	0.86	0.61	0.382	0.505	0.0070
57	Citrulline	84.5 ± 43.3	70.5 ± 16.3	62.9 ± 9.3	73.2 ± 20.9	0.83	0.74	0.87	0.878	0.279	0.959
58	1-Methylhistidine	0.65 ± 0.21	0.63 ± 0.17	0.69 ± 0.10	0.74 ± 0.30	0.96	1.06	1.13	0.878	0.959	0.878
59	Histidine	9.4 ± 1.8	8.2 ± 0.8	7.6 ± 1.4	7.4 ± 0.7	0.87	0.81	0.79	0.130	0.038	0.0070
60	3-Methylhistidine	1.7 ± 0.2	1.5 ± 0.3	1.8 ± 0.4	2.5 ± 0.6	0.93	1.08	1.53	0.645	0.959	0.0047
61	N^G-Methylarginine	0.39 ± 0.08	0.33 ± 0.07	0.27 ± 0.08	0.12 ± 0.02	0.85	0.69	0.30	0.235	0.010	0.00016
62	Lysine	144.9 ± 42.6	101.0 ± 29.2	122.3 ± 26.0	143.3 ± 27.8	0.70	0.84	0.99	0.038	0.382	0.721
63	Ornithine	10.8 ± 6.6	12.4 ± 6.7	8.4 ± 7.0	13.2 ± 8.9	1.14	0.78	1.22	0.721	0.235	0.798
64	Arginine	19.6 ± 10.6	13.9 ± 9.7	20.7 ± 7.1	16.3 ± 11.4	0.71	1.06	0.83	0.328	0.721	0.442
65	Pyroglutamic acid	2.8 ± 0.9	3.5 ± 1.1	3.1 ± 1.1	3.3 ± 1.1	1.24	1.10	1.16	0.442	0.721	0.505
**Kynurenine pathway metabolite**											
66	Picolinic acid	0.014 ± 0.004	0.016 ± 0.004	0.015 ± 0.003	0.011 ± 0.005	1.18	1.06	0.82	0.195	0.798	0.105
67	Quinolinic acid	0.074 ± 0.074	0.062 ± 0.014	0.044 ± 0.013	0.13 ± 0.06	0.84	0.59	1.80	0.235	0.505	0.015
68	Serotonin	0.14 ± 0.14	0.14 ± 0.13	0.033 ± 0.021	0.17 ± 0.15	0.99	0.23	1.15	0.959	0.065	1.000
69	Kynurenine	0.46 ± 0.10	0.66 ± 0.23	0.56 ± 0.16	0.66 ± 0.21	1.44	1.22	1.45	0.130	0.279	0.050
70	Kynurenic acid	0.0091 ± 0.0045	0.0098 ± 0.0017	0.0074 ± 0.0021	0.0107 ± 0.0051	1.08	0.81	1.17	0.195	0.798	0.505
71	5-Hydroxyindoleacetic acid	0.046 ± 0.014	0.050 ± 0.013	0.040 ± 0.006	0.052 ± 0.015	1.10	0.88	1.13	0.574	0.645	0.505
72	Anthranilic acid	0.0027 ± 0.0008	0.0044 ± 0.0011	0.0037 ± 0.0013	0.0027 ± 0.0008	1.62	1.35	1.00	0.0047	0.195	0.798
**Nucleoside**											
73	5,6-Dihydrouridine	1.08 ± 0.18	0.96 ± 0.21	1.08 ± 0.29	1.0 ± 0.6	0.89	1.00	0.94	0.279	0.798	0.328
74	Pseudouridine	0.61 ± 0.15	0.53 ± 0.12	0.57 ± 0.17	0.62 ± 0.37	0.87	0.93	1.01	0.161	0.645	0.328
75	Cytidine	0.88 ± 0.12	0.75 ± 0.06	0.65 ± 0.06	0.79 ± 0.09	0.85	0.74	0.90	0.038	0.0047	0.130
76	5-Methylcytidine	0.75 ± 0.17	0.63 ± 0.10	0.67 ± 0.13	0.78 ± 0.17	0.83	0.89	1.04	0.105	0.442	0.798
77	N^2,N^2-Dimethylguanosine	0.0064 ± 0.0006	0.0061 ± 0.0003	0.0061 ± 0.0016	0.0087 ± 0.0019	0.95	0.96	1.35	0.442	0.065	0.010
78	1-Methyladenosine	0.0008 ± 0.0002	0.0007 ± 0.0001	0.0007 ± 0.0002	0.0008 ± 0.0003	0.82	0.88	0.99	0.065	0.279	0.328
79	N^6-Methyladenosine	0.0008 ± 0.0002	0.0007 ± 0.0001	0.0008 ± 0.0002	0.0008 ± 0.0003	0.83	0.95	1.00	0.038	0.878	0.574
80	MTA	0.0050 ± 0.0018	0.0034 ± 0.0029	0.0031 ± 0.0017	0.0049 ± 0.0045	0.67	0.62	0.96	0.235	0.050	0.161

[a]Values normalized to the corresponding CONTROL concentration values

[b]P-value evaluated by Wilcoxon rank-sum test

Table S3. Levels and P-values of metabolites in plasma of female rat models

No.	Metabolite	Concentration (ng/μL, Mean ± SD)				Normalized value[a]			P-value[b]		
		Control	FR	HF	OB	Control vs FR	Control vs HF	Control vs OB	Control vs FR	Control vs HF	Control vs OB
**Organic acid**											
1	Pyruvic acid	12.7 ± 9.0	8.4 ± 8.9	22.2 ± 24.2	11.9 ± 14.6	0.66	1.75	0.94	0.710	0.749	0.710
2	Acetoacetic acid	30.1 ± 8.6	35.6 ± 6.5	30.1 ± 7.6	32.2 ± 5.5	1.18	1.00	1.07	0.456	1.000	0.902
3	Lactic acid	189.4 ± 84.4	156.0 ± 50.6	237.5 ± 138.6	154.4 ± 65.0	0.82	1.25	0.82	0.535	0.710	0.535
4	Glycolic acid	2.8 ± 1.6	1.7 ± 0.7	1.6 ± 1.0	3.7 ± 2.5	0.60	0.57	1.33	0.383	0.209	0.620
5	Phenylacetic acid	0.19 ± 0.04	0.19 ± 0.03	0.23 ± 0.07	0.17 ± 0.02	0.99	1.22	0.91	1.000	0.209	0.535
6	Oxalic acid	2.6 ± 0.9	4.5 ± 3.7	2.6 ± 1.5	3.2 ± 1.0	1.71	1.00	1.23	0.456	0.620	0.383
7	2-Hydroxybutyric acid	2.3 ± 1.3	2.5 ± 0.8	2.8 ± 1.0	6.5 ± 1.4	1.09	1.23	2.85	0.805	0.383	0.0012
8	3-Hydroxypropionic acid	0.42 ± 0.06	0.51 ± 0.10	0.55 ± 0.11	0.47 ± 0.14	1.21	1.31	1.12	0.128	0.053	0.535
9	3-Hydroxybutyric acid	61.8 ± 35.9	66.1 ± 25.5	64.1 ± 33.5	169.3 ± 76.8	1.07	1.04	2.74	0.620	0.902	0.011
10	2-Hydroxyisovaleric acid	0.27 ± 0.06	0.32 ± 0.06	0.30 ± 0.05	0.39 ± 0.07	1.22	1.14	1.46	0.128	0.456	0.017
11	Succinic acid	2.4 ± 1.2	2.3 ± 0.7	2.6 ± 0.7	2.5 ± 0.6	0.96	1.11	1.04	0.805	0.535	1.000
12	Fumaric acid	0.47 ± 0.25	0.34 ± 0.14	0.58 ± 0.30	0.52 ± 0.17	0.72	1.22	1.09	0.383	0.710	0.535
13	α-Ketoglutaric acid	7.1 ± 2.6	5.5 ± 0.7	7.0 ± 2.2	7.8 ± 1.8	0.77	0.99	1.09	0.165	0.902	0.456
14	Hippuric acid	1.1 ± 0.7	1.2 ± 0.6	1.1 ± 0.3	0.23 ± 0.04	1.16	1.06	0.21	0.535	0.710	0.00058
15	Malic acid	6.4 ± 0.9	6.4 ± 0.7	6.9 ± 0.9	6.6 ± 0.6	1.00	1.08	1.03	0.805	0.456	1.000
16	3-Indolepropionic acid	0.74 ± 0.29	0.45 ± 0.17	0.49 ± 0.17	0.18 ± 0.02	0.61	0.66	0.24	0.073	0.165	0.00058
17	*cis*-Aconitic acid	0.077 ± 0.053	0.085 ± 0.020	0.100 ± 0.061	0.11 ± 0.04	1.10	1.30	1.39	0.710	0.710	0.209
18	3-Indolecarboxylic acid	0.094 ± 0.004	0.095 ± 0.005	0.089 ± 0.005	0.093 ± 0.005	1.01	0.95	0.99	0.805	0.073	0.710
19	3-Indolelactic acid	0.12 ± 0.05	0.08 ± 0.02	0.09 ± 0.03	0.10 ± 0.04	0.69	0.82	0.86	0.209	0.620	0.535
20	Citric acid	9.4 ± 2.8	9.1 ± 1.1	8.2 ± 1.8	12.4 ± 3.8	0.97	0.87	1.32	1.000	0.456	0.165
21	Isocitric acid	1.7 ± 0.9	1.6 ± 0.6	1.4 ± 0.5	2.1 ± 0.9	1.00	0.84	1.30	0.535	0.902	0.318
**Fatty acid**											
22	Myristic acid	1.5 ± 0.5	1.2 ± 0.8	0.9 ± 0.6	2.4 ± 0.9	0.77	0.58	1.58	0.383	0.038	0.259
23	Palmitoleic acid	5.5 ± 2.4	4.5 ± 4.0	2.8 ± 2.7	8.1 ± 4.4	0.83	0.51	1.49	0.456	0.073	0.259
24	Palmitic acid	73.1 ± 14.6	67.5 ± 13.6	60.1 ± 19.2	85.0 ± 16.9	0.92	0.82	1.16	0.535	0.209	0.318

25	Linoleic acid	87.9 ± 11.9	82.5 ± 14.3	76.6 ± 15.9	74.9 ± 23.2	0.94	0.87	0.85	0.620	0.209	0.318
26	Oleic acid	34.6 ± 12.6	26.4 ± 14.8	21.6 ± 13.9	59.4 ± 21.8	0.76	0.63	1.72	0.318	0.073	0.073
27	α-Linolenic acid	3.5 ± 1.8	2.8 ± 0.8	2.7 ± 1.1	3.5 ± 2.1	0.81	0.79	0.99	0.620	0.456	0.805
28	Stearic acid	61.2 ± 9.1	64.8 ± 11.5	53.8 ± 8.8	70.7 ± 10.8	1.06	0.88	1.15	0.710	0.209	0.259
29	Arachidonic acid	115.4 ± 14.7	128.9 ± 21.6	111.3 ± 19.8	120.1 ± 23.1	1.12	0.96	1.04	0.383	1.000	0.620
30	Eicosadienoic acid	1.7 ± 0.6	2.0 ± 0.5	1.7 ± 0.4	2.3 ± 0.5	1.19	1.00	1.38	0.318	0.805	0.097
31	Gondoic acid	0.46 ± 0.15	0.43 ± 0.04	0.38 ± 0.04	0.62 ± 0.11	0.92	0.82	1.33	0.710	0.318	0.053
32	Docosatetraenoic acid	21.5 ± 5.4	26.3 ± 5.8	20.5 ± 2.7	34.5 ± 8.9	1.22	0.95	1.60	0.165	0.902	0.011
**Amino acid**											
33	Creatinine	1.6 ± 0.3	1.4 ± 0.1	1.5 ± 0.2	1.5 ± 0.3	0.90	0.95	0.98	0.318	0.620	0.805
34	Phenylalanine	11.9 ± 1.3	12.5 ± 1.8	12.1 ± 1.3	10.5 ± 1.2	1.05	1.01	0.88	0.383	0.902	0.073
35	Tryptophan	11.1 ± 1.4	10.0 ± 1.2	10.6 ± 1.3	11.1 ± 1.5	0.90	0.96	1.00	0.128	0.535	0.902
36	Leucine	21.0 ± 4.9	26.4 ± 5.4	21.0 ± 4.4	18.5 ± 2.6	1.25	1.00	0.88	0.073	1.000	0.535
37	Isoleucine	12.2 ± 1.6	16.4 ± 2.3	13.0 ± 2.2	12.5 ± 1.4	1.34	1.06	1.02	0.0070	0.535	0.710
38	Methionine	7.0 ± 0.9	7.6 ± 0.6	7.1 ± 1.0	6.4 ± 0.8	1.09	1.03	0.92	0.128	1.000	0.456
39	Proline	15.7 ± 1.4	18.0 ± 2.2	17.2 ± 3.0	15.9 ± 2.6	1.15	1.10	1.01	0.026	0.318	0.620
40	Pipecolic acid	0.37 ± 0.08	0.46 ± 0.14	0.55 ± 0.12	0.33 ± 0.13	1.25	1.50	0.90	0.318	0.0041	0.535
41	Tyrosine	15.2 ± 2.1	15.9 ± 1.7	15.9 ± 2.9	12.1 ± 2.6	1.05	1.05	0.80	0.710	0.805	0.053
42	Valine	23.2 ± 2.9	30.6 ± 4.9	24.5 ± 4.2	22.1 ± 2.9	1.32	1.06	0.95	0.017	0.535	0.620
43	α-Aminobutyric acid	2.0 ± 1.0	2.3 ± 1.2	2.1 ± 1.0	4.3 ± 1.2	1.19	1.07	2.16	0.710	0.902	0.0041
44	Alanine	45.2 ± 8.4	49.9 ± 9.2	53.1 ± 9.3	37.9 ± 8.1	1.11	1.18	0.84	0.259	0.165	0.165
45	4-Hydroxyproline	1.5 ± 0.2	1.5 ± 0.2	1.4 ± 0.1	1.3 ± 0.2	0.97	0.91	0.85	0.535	0.097	0.038
46	Homoserine	25.8 ± 2.4	33.1 ± 5.5	27.8 ± 3.6	40.7 ± 13.5	1.28	1.08	1.58	0.0070	0.165	0.017
47	Creatine	37.6 ± 5.0	40.4 ± 4.0	45.2 ± 6.2	23.1 ± 8.0	1.07	1.20	0.61	0.383	0.026	0.0041
48	Threonine	197.5 ± 77.6	262.1 ± 82.8	249.5 ± 66.6	284.1 ± 82.7	1.33	1.26	1.44	0.259	0.259	0.073
49	α-Aminoadipic acid	0.19 ± 0.05	0.14 ± 0.02	0.18 ± 0.06	0.20 ± 0.04	0.75	0.93	1.05	0.128	0.535	1.000
50	Glycine	16.0 ± 3.4	17.9 ± 4.1	15.0 ± 3.2	12.8 ± 3.6	1.12	0.94	0.80	0.456	0.535	0.209
51	Glutamic acid	13.5 ± 2.6	13.7 ± 2.9	13.3 ± 2.4	13.7 ± 3.9	1.01	0.99	1.01	1.000	0.805	0.805
52	β-Alanine	0.24 ± 0.04	0.22 ± 0.05	0.26 ± 0.05	0.28 ± 0.05	0.95	1.09	1.20	0.805	0.456	0.073
53	Serine	20.9 ± 1.9	24.2 ± 3.6	20.5 ± 2.0	23.9 ± 2.6	1.16	0.98	1.14	0.097	0.805	0.073
54	Glutamine	62.8 ± 5.9	61.0 ± 6.7	61.8 ± 9.4	61.1 ± 6.8	0.97	0.98	0.97	0.620	0.805	0.710
55	Asparagine	7.5 ± 1.8	8.5 ± 1.5	8.3 ± 2.2	7.0 ± 1.6	1.13	1.11	0.93	0.456	0.383	0.902
56	Aspartic acid	0.89 ± 0.37	0.85 ± 0.14	0.75 ± 0.30	0.61 ± 0.15	0.95	0.84	0.68	1.000	0.710	0.209
57	Citrulline	55.4 ± 8.7	64.2 ± 18.0	55.9 ± 16.3	41.9 ± 10.7	1.16	1.01	0.76	0.535	0.710	0.017
58	1-Methylhistidine	0.40 ± 0.07	0.40 ± 0.09	0.37 ± 0.04	0.39 ± 0.09	1.01	0.93	0.98	0.902	0.535	1.000
59	Histidine	6.4 ± 0.9	7.3 ± 1.4	6.8 ± 1.1	6.0 ± 0.7	1.15	1.08	0.94	0.165	0.383	0.620
60	3-Methylhistidine	1.00 ± 0.24	1.0 ± 0.2	1.1 ± 0.1	1.3 ± 0.3	1.00	1.06	1.31	1.000	0.805	0.165
61	N^G-Methylarginine	0.26 ± 0.08	0.38 ± 0.14	0.33 ± 0.08	0.13 ± 0.03	1.44	1.25	0.51	0.097	0.318	0.0023
62	Lysine	125.5 ± 35.2	155.9 ± 39.8	179.8 ± 44.6	189.5 ± 49.2	1.24	1.43	1.51	0.259	0.0070	0.038
63	Ornithine	9.5 ± 7.8	12.4 ± 9.6	11.8 ± 7.9	7.5 ± 3.9	1.31	1.25	0.79	0.710	0.456	1.000
64	Arginine	14.5 ± 5.7	15.0 ± 10.5	15.2 ± 7.0	14.1 ± 5.9	1.04	1.05	0.97	0.805	0.535	1.000
65	Pyroglutamic acid	3.4 ± 1.0	3.9 ± 1.7	3.2 ± 1.3	2.9 ± 1.2	1.14	0.95	0.84	0.710	0.805	0.383
**Kynurenine pathway metabolite**											
66	Picolinic acid	0.016 ± 0.004	0.021 ± 0.008	0.016 ± 0.005	0.012 ± 0.004	1.30	0.99	0.78	0.209	0.710	0.128
67	Quinolinic acid	0.054 ± 0.028	0.047 ± 0.017	0.046 ± 0.020	0.11 ± 0.03	0.87	0.85	2.05	0.620	0.620	0.0070
68	Serotonin	0.085 ± 0.037	0.096 ± 0.035	0.090 ± 0.042	0.06 ± 0.03	1.12	1.05	0.72	0.620	0.902	0.535
69	Kynurenine	0.37 ± 0.13	0.48 ± 0.06	0.46 ± 0.13	0.41 ± 0.13	1.31	1.26	1.12	0.128	0.209	0.383
70	Kynurenic acid	0.011 ± 0.002	0.014 ± 0.004	0.011 ± 0.002	0.010 ± 0.003	1.22	0.99	0.90	0.456	1.000	0.456
71	5-Hydroxyindoleacetic acid	0.082 ± 0.017	0.085 ± 0.009	0.086 ± 0.016	0.10 ± 0.02	1.04	1.05	1.20	0.620	0.710	0.128
72	Anthranilic acid	0.0041 ± 0.0020	0.0056 ± 0.0011	0.0053 ± 0.0009	0.0037 ± 0.0017	1.36	1.28	0.91	0.128	0.209	0.902
**Nucleoside**											
73	5,6-Dihydrouridine	1.6 ± 0.3	1.9 ± 0.3	1.7 ± 0.3	1.7 ± 0.3	1.20	1.09	1.10	0.165	0.383	0.456
74	Pseudouridine	0.71 ± 0.13	0.81 ± 0.08	0.72 ± 0.07	0.75 ± 0.10	1.14	1.01	1.05	0.209	1.000	0.620
75	Cytidine	0.66 ± 0.09	0.69 ± 0.20	0.67 ± 0.16	0.61 ± 0.10	1.05	1.03	0.93	0.620	0.902	0.456
76	5-Methylcytidine	0.59 ± 0.05	0.56 ± 0.04	0.59 ± 0.06	0.78 ± 0.09	0.95	1.00	1.33	0.383	0.902	0.0012
77	N^2,N^2-Dimethylguanosine	0.0064 ± 0.0009	0.0099 ± 0.0033	0.0101 ± 0.0047	0.012 ± 0.0049	1.54	1.58	1.80	0.128	0.383	0.0012
78	1-Methyladenosine	0.0010 ± 0.0001	0.0010 ± 0.0002	0.0009 ± 0.0002	0.0013 ± 0.0003	1.04	0.95	1.35	0.902	1.000	0.026
79	N^6-Methyladenosine	0.0010 ± 0.0001	0.0011 ± 0.0002	0.0009 ± 0.0002	0.0013 ± 0.0003	1.05	0.94	1.30	0.710	1.000	0.053
80	MTA	0.0029 ± 0.0017	0.0025 ± 0.0019	0.0036 ± 0.0025	0.0047 ± 0.0019	0.86	1.23	1.59	0.620	0.805	0.053

[a]Values normalized to the corresponding CTRL concentration values

[b]P-value calculated by Wilcoxon rank-sum test

Table S4. Composition of the standard and 45% high-fat diets

Content	Standard diet Kcal (%)	45% high-fat diet Kcal (%)
Carbohydrate	59	35
Protein	27	20
Fat	14	45
**total**	**100**	**100**

Standard diet (altromin, 1314) and High-fat diet (research diets, D12451)
